# Postmortem Metabolomics: Strategies to Assess Time-Dependent Postmortem Changes of Diazepam, Nordiazepam, Morphine, Codeine, Mirtazapine and Citalopram

**DOI:** 10.3390/metabo11090643

**Published:** 2021-09-20

**Authors:** Lana Brockbals, Yannick Wartmann, Dylan Mantinieks, Linda L. Glowacki, Dimitri Gerostamoulos, Thomas Kraemer, Andrea E. Steuer

**Affiliations:** 1 Department of Forensic Pharmacology and Toxicology, Zurich Institute of Forensic Medicine, University of Zurich, Winterthurerstrasse 190/52, 8057 Zurich, Switzerland; lana.brockbals@irm.uzh.ch (L.B.); yannick.wartmann@irm.uzh.ch (Y.W.); thomas.kraemer@irm.uzh.ch (T.K.); 2 Department of Forensic Medicine, Monash University, 65 Kavanagh Street, Southbank, VIC 3006, Australia; dylan.mantinieks@vifm.org (D.M.); dimitri.gerostamoulos@vifm.org (D.G.); 3 Victorian Institute of Forensic Medicine, 65 Kavanagh Street, Southbank, VIC 3006, Australia; linda.glowacki@vifm.org

**Keywords:** postmortem metabolomics, time-dependent postmortem redistribution, prediction strategies, mathematical modeling, correlation analysis

## Abstract

Postmortem redistribution (PMR) can result in artificial drug concentration changes following death and complicate forensic case interpretation. Currently, no accurate methods for PMR prediction exist. Hence, alternative strategies were developed investigating the time-dependent postmortem behavior of diazepam, nordiazepam, morphine, codeine, mirtazapine and citalopram. For 477 authentic postmortem cases, femoral blood samples were collected at two postmortem time-points. All samples were quantified for drugs of abuse (targeted; liquid chromatography-tandem mass spectrometry LC-MS/MS) and characterized for small endogenous molecules (untargeted; gas chromatography-high resolution MS (GC-HRMS). Trends for significant time-dependent concentration decreases (diazepam (*n* = 137), nordiazepam (*n* = 126)), increases (mirtazapine (*n* = 55), citalopram (*n* = 50)) or minimal median postmortem changes (morphine (*n* = 122), codeine (*n* = 92)) could be observed. Robust mathematical mixed effect models were created for the generalized postmortem behavior of diazepam and nordiazepam, which could be used to back-calculate drug concentrations towards a time-point closer to the estimated time of death (caution: inter-individual variability). Significant correlations between time-dependent concentration changes of morphine, mirtazapine and citalopram with individual endogenous molecules could be determined; no correlation was deemed strong enough for successful a posteriori estimation on the occurrence of PMR for specific cases. The current dataset did successfully lead to a significant knowledge gain in further understanding the time-dependent postmortem behavior of the studied drugs (of abuse).

## 1. Introduction

While it is important in a forensic medical investigation to accurately identify and quantify drugs (of abuse), postmortem redistribution (PMR) processes can lead to artificial postmortem drug concentration changes in the deceased body over time which can complicate forensic case interpretation [1]; postmortem phenomena include passive diffusion processes, degradation or drug neo-formation (e.g., driven by microorganisms) [2]. Underlying mechanisms of PMR are not fully understood to date, but general drug properties (e.g., volume of distribution, lipophilicity and protein binding affinity) and biochemical processes (antemortem and postmortem factors as well as a potential agonal phase) are thought to be significant contributing factors [2,3]. Over the years, several attempts have been made trying to predict the occurrence of PMR in order to better interpret postmortem drug concentrations. The most commonly used tools for prediction are the cardiac-to-femoral blood concentration ratio (C/P-ratio) along with the liver-to-femoral blood concentration ratio (L/P-ratio). However, neither of these ratios have been found to demonstrate a clear relationship to a drugs’ physicochemical properties that are, as mentioned above, thought to influence the occurrence/extent of PMR. Thus, the prediction power of such approaches is very limited [4,5,6]. In addition, PMR of some drugs (of abuse) was previously modelled by quantitative structure-activity relationship (QSAR) methods. While these helped to understand contributing molecular, physicochemical and structural properties for the occurrence of PMR, the complexity and time-dependent nature of PMR omitted accurate prediction of the degree of redistribution [7,8]. Indeed, time-dependent postmortem concentration changes of several drugs (of abuse) have previously been described in a limited number of small case sets. Using authentic human cases, drug concentrations of peripheral blood samples between mortuary admission and autopsy or antemortem and mortuary admission have previously been compared [9,10,11]. Additionally, a computed-tomography-guided biopsy sampling tool has previously been utilized for minimal invasive tissue and body fluid collection several hours before the medico-legal autopsy and drug concentration results compared to autopsy samples [12,13,14,15]. All these studies support the fact that PMR also occurs in peripheral specimens (e.g., femoral blood) and understanding time-dependent postmortem concentration changes is crucial for reliable forensic case interpretation. While these allow a general trend to be predicted for the time-dependent postmortem behavior of certain drugs and drug classes, an individual estimation on the occurrence of PMR on a case-by-case basis is not possible due to large inter-individual variabilities. As an alternative, Langford and Pounder proposed the general concept of postmortem biochemical changes in blood possibly paralleling drug redistribution mechanisms [16]. An endogenous molecule/feature that shows a strong correlation with a drug (of abuse) or drug class could be used as a surrogate marker to further study time-dependent PMR and a posteriori estimation of PMR occurrence could be attempted. A recent proof-of-concept study took up this idea and utilized an untargeted gas chromatography-high resolution mass spectrometry (GC-HRMS) metabolomics workflow to find individual statistically significant correlations between the time-dependent concentration changes of morphine/methadone and some endogenous molecules/features [17]. As this study was only comprised of a small case set (morphine: *n* = 19; methadone: *n* = 11), robustness and reproducibility were still needed to be confirmed, which was one of the aims of the current study, utilizing 477 authentic postmortem cases, where femoral blood samples at two postmortem time points were available. This uniquely large case set was also used to describe the time-dependent postmortem concentration changes of diazepam (*n* = 137), nordiazepam (*n* = 126), morphine (*n* = 122), codeine (*n* = 92), mirtazapine (*n* = 55) and citalopram (*n* = 50) and mathematical modelling of their generalized postmortem behavior in an attempt to determine a potential back-calculation towards a time-point closer to the time of death. 

## 2. Results and Discussion

### 2.1. Storage and Shipping

In order to exclude a significant influence of the storage conditions and/or shipment on the presented study, quantification results of 90 randomly selected cases were compared in a first step. The selected reference points were the drug concentrations of diazepam, nordiazepam, morphine, codeine, mirtazapine and citalopram obtained during routine analysis at the Victorian Institute of Forensic Medicine (VIFM) for mortuary admission samples (t1) utilizing a high throughput method for semi-quantification of 327 drugs in blood [18]. These were compared to quantification results at the Zurich Institute of Forensic Medicine (ZIFM) after storage and shipment as detailed in the materials and methods section [19]. Direct visual comparison and Bland-Altman analyses for all six drugs (of abuse) did not show significant concentration-dependent differences between the two analytical methods (for details refer to Figures S1 and S2 in the Supplementary Material). Mirtazapine concentrations measured at the ZIFM were constantly lower compared to those obtained at the VIFM, which might indicate calibration differences between the two methods or, more unlikely, constant drug instability. However, these do not appear to be relevant for the current study, as they also do not show concentration dependency. Hence, storage and shipping conditions do not seem to have a significant influence on the presented study and it is assumed that the following results are due to genuine postmortem changes and not significantly altered by storage and shipping conditions. Only quantification results from the ZIFM were used for the following interpretation of results. Stability of endogenous substances could not be assessed before and after shipment due to the lack of data. Previous studies e.g., suggest ongoing metabolism in biofluids after collection, particularly at room temperature [20,21]. However, within the current study, to overcome confounders based on different storage times, only samples from the same individual (t1 and t2) were directly compared to each other (assumed to show the same stability pattern) and only case-specific concentration changes were afterwards compared between other individuals, rather than direct comparison across all cases. 

### 2.2. Time-Dependent Concentration Changes of Drugs (of Abuse)

In general, for all six studied drugs (of abuse), drug concentration decreases as well as increases were observed. Therefore, while in the following paragraphs the authors attempted to describe the generalizing behavior of the individual drugs (of abuse), it is crucial for forensic toxicologists to be aware of the inter-individual variability between cases to be taken into account for interpretation.

**Figure 1:**
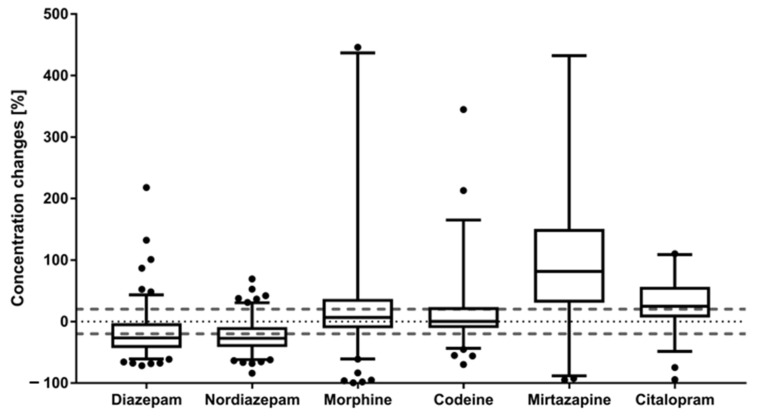
Percentage concentration changes are displayed for the six studied drugs (of abuse) in box-whisker plots; whiskers indicate the 5–95% percentile; within the boxes, the median is displayed; y-axis was cut at 500% for better visibility, but cases above 500% concentration changes were included for the statistics (above 500%: morphine: *n* = 5; codeine: *n* = 2; mirtazapine: *n* = 2; citalopram: *n* = 2); dotted line indicates 0% change; dashed lines indicate ±20% change region.

Both diazepam (*n* = 137) and nordiazepam (*n* = 126) showed a median concentration decrease of −27% between t1 and t2 across all analyzed cases as visualized in Figure 1 (diazepam: min −72%, max +220%; nordiazepam: min −84%, max +69%). Such observed moderate time-dependent concentration decreases are in line with current literature; e.g., Gerostamoulos et al. previously found a median autopsy/mortuary admission ratio of 0.82 and 0.91 for diazepam (*n* = 53) and nordiazepam (*n* = 57), respectively [9]. Similarly, comparing antemortem with postmortem benzodiazepine concentrations, Mantinieks et al. reported minor concentration decreases for this drug class (*n* = 135) [11]. As proposed by Lemaire et al., negative concentration changes in femoral blood over time may be the consequence of postmortem microbial degradation (*n* = 24) [6]. 

For morphine (*n* = 122) and codeine (*n* = 92), no significant postmortem concentration changes were observed for median values (morphine: median +6.5%; codeine: median +0.2%; Figure 1). Other time-dependent studies have previously also concluded a negligible trend for PMR based on no significant mean concentration differences between mortuary admission and autopsy femoral blood samples for both codeine (*n* = 34) and morphine (*n* = 11 and *n* = 40) [9,22]. Similarly, Hargrove and Molina found no significant postmortem redistribution of morphine (*n* = 18) from central sites within the first 24 h after death [23]. However, in contrast, a small case study (*n* = 12) by Staeheli et al. found significant increases of morphine concentrations in femoral blood between two postmortem time-points [13]. This disagreement between the studies is most likely based on extreme inter-individual variabilities that are also observed within the current study; ranges of time-dependent concentration changes between −99% to +7000% for morphine cases and −70% to 1700% for codeine cases. Similar variability between cases was also observed by Mantinieks et al., who found postmortem to antemortem concentration ratios of 0.04 to 122 for morphine cases (*n* = 204) and 0.02 to 9.1 for codeine cases (*n* = 52). This indicates that in their study an individual case showed a postmortem morphine concentration 122 times higher than the previous antemortem concentration, although extreme differences due to therapeutic administration of morphine by healthcare workers could not be excluded [11]. Further, Langford et al. found morphine concentration changes between −83% to +166% (median +56%) between antemortem and postmortem drug concentrations (*n* = 11) [24]. Tolerance effects could be a contributing factor to such inter-individual differences. Previously proposed redistribution mechanisms for morphine are passive diffusion processes from muscle tissue into the femoral blood stream and the hydrolytic cleavage of morphine glucuronides back to morphine [2,13]. The latter, however, albeit possible, was deemed unlikely due to the relatively short time-frame between time of death and sample collection in this study [13,25,26]. 

In contrast, the two studied antidepressants mirtazapine (*n* = 55) and citalopram (*n* = 50) showed a trend for time-dependent concentration increases with a median of +82% and +25%, respectively as displayed in Figure 1 (mirtazapine: min −95%, max +960%; citalopram: min −95%, max +3900%). For mirtazapine, these results agree with two smaller time-dependent studies, where in one study average mirtazapine concentration increases between 20 and 50% were observed (*n* = 14) [9], whereas in the other study, a range of concentration changes between −15 to +41% (mean +12%, median +5%) were found across seven cases [15]. For citalopram, a set of 10 cases showed a range of time-dependent concentration changes between −50% and +34% (median −13%), indicating a trend towards time-dependent concentration decreases [15]. A second study (*n* = 13), however, found average citalopram concentration increases between 20 to 50%, similar to the current results [9]. As the latter study is based on the same sample collection workflow at the VIFM, this is not surprising. Generally, time-dependent concentration increases as observed for mirtazapine and citalopram are often thought to be driven by passive diffusion processes e.g., from central organs into its surroundings or from fatty/muscle tissue into the peripheral blood [2].

For all six studied drugs (of abuse), no direct correlation of concentration changes with the case specific pre-autopsy interval could be determined. Large drug concentration increases/decreases were found for both short and extensive pre-autopsy intervals. For example, cases 2658 and 2677 both showed a diazepam concentration decrease of −68% between t1 and t2. The corresponding pre-autopsy intervals, however, were 29 h and 163 h, respectively. Potential reasons behind this postmortem behavior could be different phases of redistribution as previously proposed for quetiapine [15]. Alternatively, it may be due to a multitude of physical and chemical processes that occur immediately after death and can lead to rapid changes in drug concentrations [27]. Due to the utilization of routine cases, however, this very early postmortem period could not be studied (i.e., the time interval between time of death and admission to the institute). Details on average timings are displayed in Table 1; case-specific intervals can be found within the supplementary material (Table S1).

### 2.3. Strategies to Predict PMR/for a Posteriori Estimation of PMR Occurrence

As detailed within the introduction, currently no accurate methods for PMR prediction and/or a posteriori estimation of PMR occurrence exist. Hence, alternative strategies were developed that should aid the interpretation of forensic toxicological postmortem cases.

#### 2.3.1. Mixed Effect Models

Utilizing the uniquely large postmortem case set of the current study it was possible for the first time to create mathematical mixed-effect models for the generalized time-dependent postmortem behavior of diazepam, nordiazepam, morphine, codeine, mirtazapine and citalopram. Compared to a fixed-effect model, this study used a mixed effect model incorporating both fixed and random effects, which allows for a generalized usage of the models. The aim was to check for robustness of the models and attempt back-calculations towards a time-point closer to the time of death or even the time of death. This could be particularly useful for cases where sample collection is only possible after an extensive postmortem interval. 

As detailed in Table 2, a negative lambda for diazepam and nordiazepam was calculated, while positive lambda values for morphine, codeine, mirtazapine and citalopram were returned upon input of the drug quantification data. Within the created mixed-effect models, lambda is a calculated factor that defines the influence of the time-variable on the drug concentration changes; a negative lambda indicates generalized time-dependent concentration decreases, whereas a positive lambda overall indicates drug concentration increases over time. This is in accordance with the aforementioned generalized time-dependent postmortem behavior of the drugs; trend for concentration decreases for diazepam and nordiazepam; median concentration increases observed for morphine, codeine, mirtazapine and citalopram. For morphine and codeine, the calculated confidence interval spans from negative to positive, including zero. This is an indication that the time variable might not have an effect on the dataset, meaning that time-dependent concentration changes might not be statistically significant for morphine and codeine [28]. Based also on the high inter-individual variability that was described previously, it was concluded that mathematical modelling of the time-dependent postmortem behavior of morphine and codeine was not successful, and this should not be used for further interpretation. The calculated confidence interval for mirtazapine and citalopram in contrast did not overlap zero, which indicates statistical significance at a first glance. To test for robustness of the model, the model creation was repeated 50 times (each time based on a random selection of 80% of the cases; mirtazapine: *n* = 44; citalopram: *n* = 40), with a new lambda calculated every time. As seen in Table 2, the relative standard deviations (RSD) of the calculated lambdas were 27 and 15% for mirtazapine and citalopram, respectively. This was deemed too large of a variation to accept the robustness of the model. Despite the seemingly large number of cases in this dataset, additional mirtazapine and citalopram cases (n > 50) are still needed for reliable creation of a mathematical model. However, in a forensic context, this seems hard to achieve. 

For diazepam and nordiazepam, the training sets of the models were comprised of 109 and 100 cases, respectively. For both drugs, the confidence interval did not span across zero and the relative standard deviation of lambda between repeated models was 7.2 and 5.4%, respectively. With these results indicating sufficient robustness of the models, the prediction accuracy of the test set was studied. For this, the quantified diazepam/nordiazepam concentrations at t2 (cases not used for the creation of the model; diazepam: *n* = 28; nordiazepam: *n* = 26) were given as input values for the model and prediction accuracy between model-calculated t1 concentration and experimentally quantified t1 concentrations was compared (with ∆t being the time-difference between t1 and t2 sample collection). Median prediction accuracies were found to be 109% for diazepam and 108% for nordiazepam (individual accuracy values are listed in Table S2 of the supplementary material). 

These results show that the general concept of modelling the time-dependent postmortem behavior of diazepam and nordiazepam with the following formulas does seem to work:

Diazepam: c(t1) = c(t2) ∗ e^(−0.00296044*(∆t)).(1)

Nordiazepam: c(t1) = c(t2) ∗ e^(−0.00303677*(∆t)).(2)

However, the two created models are not without limitations, which should carefully be evaluated before use. Firstly, the models are based on time-dependent concentration changes between mortuary admission and autopsy samples with a mean pre-admission interval of 13 h for diazepam cases and 12 h for nordiazepam cases; the quickest mortuary admission sample was collected 1.8 h after estimated time of death (t0 − t1), while the longest pre-autopsy interval was 383 h for both drugs (of abuse) (t0 − t2). Generally, the use of a mathematical mixed effect model allows for extrapolation of the data outside the limits of the used training data by additionally incorporating random effects [28]. Hence, usage of the model for cases with a pre-admission interval < 1.8 h or for pre-autopsy intervals > 383 h would be possible from a mathematical point of view. However, from previous experimental studies it is known that particularly within the first few minutes/hours after death a multitude of biochemical processes start that can lead to significant drug concentration changes immediately after death [27,29]. As mentioned above, the current sample collection workflow is blind to those rapid postmortem changes. Therefore, albeit very useful for forensic case interpretation, the mixed-effect model should only be used to back-calculate a drug concentration towards a time-point closer to the estimated time of death rather than actual time of death to conform to the given time intervals. Secondly, it should be stressed that the model generalizes the time-dependent postmortem behavior of diazepam and nordiazepam across the available case set and does not account for inter-individual variability. Despite very good median prediction accuracies, individual back-calculated t1 concentrations within the test set differed significantly from the quantification results (prediction accuracy ranges diazepam: 22–246%; nordiazepam: 45–169%). Following this, accurate prediction for an individual case using the proposed model might not be possible and should not be attempted for medical-legal purposes. However, it might give a general indication on the extent of diazepam/nordiazepam time-dependent postmortem concentration decreases particularly for cases with an extensive postmortem interval until sampling.

#### 2.3.2. Correlations with Endogenous Metabolites

Following the proposition of Langford and Pounder on the general possibility that postmortem biochemical changes in blood might parallel drug redistribution mechanisms and a recently published successful proof-of-concept study, the aim was to find and confirm strong positive or negative correlations between time-dependent concentration changes of drugs and endogenous metabolites in a large dataset [16,17]. These could help to further understand the underlying biochemical processes of PMR and might be used for a posteriori estimation on the occurrence of PMR for specific cases, circumventing the problem of generalized models as detailed above. Correlating endogenous molecules/features with time-dependent concentration changes of diazepam/nordiazepam/morphine/codeine/ mirtazapine and/citalopram are listed in Table 3 including the correlation coefficients and significance of the findings (*p*-value, false discovery rate (FDR) corrected; listed are all significant endogenous molecules/features and/or top correlating analytes). Results from both the targeted and untargeted data processing workflow were combined and metabolite identification was classified according to the minimum reporting standards for chemical analysis from the chemical analysis working group of the metabolomics standards initiative [30]. Following this, four levels of metabolite identification are distinguished. Level 1 includes fully identified compounds (e.g., confirmed by reference material); level 2 is comprised of putatively annotated compounds (e.g., spectral similarities with public/commercial spectral libraries); grouped as level 3 are features with putatively characterization as a compound class (e.g., spectral similarities to known compounds of a chemical class) and all unknown compounds are listed as level 4. 

**Figure 2:**
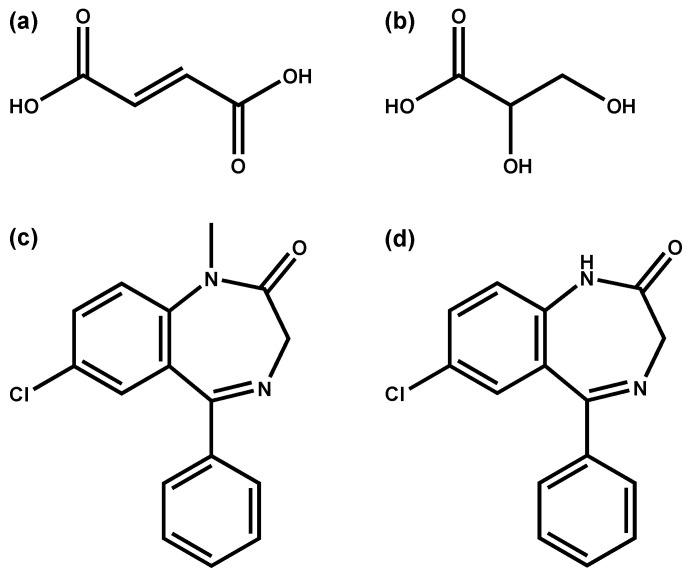
Chemical structures of (**a**) fumaric acid, (**b**) glyceric acid, (**c**) diazepam and (**d**) nordiazepam.

None of the correlations found for diazepam and nordiazepam were deemed significant (i.e., not >0.5 or <−0.5 with *p* < 0.05). Only four and five endogenous molecules/features were found to correlate with the time-dependent postmortem behavior of diazepam and nordiazepam, respectively, in a weak to moderate manner (correlation coefficients between −0.38 and 0.38; Table 3). Fumaric acid and glyceric acid showed the strongest positive and negative correlation, respectively, for both diazepam and nordiazepam. Based on their very similar chemical structure (see Figure 2) and similar pKa (around 3), one might propose a similar postmortem behavior; however, the contrary seems to be the case with fumaric acid showing a positive correlation with diazepam and nordiazepam while for glyceric acid a negative correlation with both drugs was observed. Overall, the fact that no real endogenous correlate for diazepam and/or nordiazepam was found might be caused by their proposed PMR mechanism. In fact, as mentioned above, time-dependent concentration decreases of diazepam and nordiazepam are likely thought to be the consequence of postmortem microbial degradation rather than redistribution processes in a more-narrow sense (e.g., diffusion processes) [6]. It seems that the current study was unsuccessful in finding an endogenous postmortem degradation marker, at least not with the utilized GC-HRMS workflow. Future studies might have more success examining drugs well known for their instability (e.g., some antipsychotics such as risperidone). 

For morphine, five endogenous molecules from the targeted workflow were found to significantly correlate with its time-dependent postmortem behavior, namely methionine, phenylalanine, valine, isoleucine and proline (Table 3). None of these amino acids did show a significant correlation with morphine concentration changes in the previously conducted proof-of-concept study [17]. Previously, significant negative correlations of morphine with creatinine, glutaric acid, hypoxanthine, fructose, pentadecanoic acid (C15:0), palmitoleic acid (C16:1), alanine and linoleic acid (C14:0) were established. The fact that none of these correlations could be confirmed within the current study indicates that previous case numbers were too small to find robust endogenous markers (proof-of-concept study: *n* = 19; current study: *n* = 122). Following this, the endogenous molecules established within the current study should be regarded as more robust and reproducible than the ones from the proof-of-concept study. Nevertheless, the calculated correlation coefficients only suggest a moderate connection between the time-dependent postmortem behavior of morphine and the endogenous molecules. The previously conducted proof-of-concept study focused on morphine and methadone. Although methadone was also included in the current study, the drug could only be detected in 29 cases; its metabolite EDDP (2-ethylidene-1,5-dimethyl-3,3-diphenylpyrrolidine) was quantified in 19 cases. Based on the morphine-results, these case numbers are too low for robust interpretation. Therefore, due to the likeliness of non-reproducibility/-robustness and albeit high numbers of correlations and/or strong correlation coefficients for both methadone and EDDP (methadone: 23 significant correlations; EDDP: correlation coefficients between −0.66842 and 0.79298), data is not shown in detail. 

**Figure 3:**
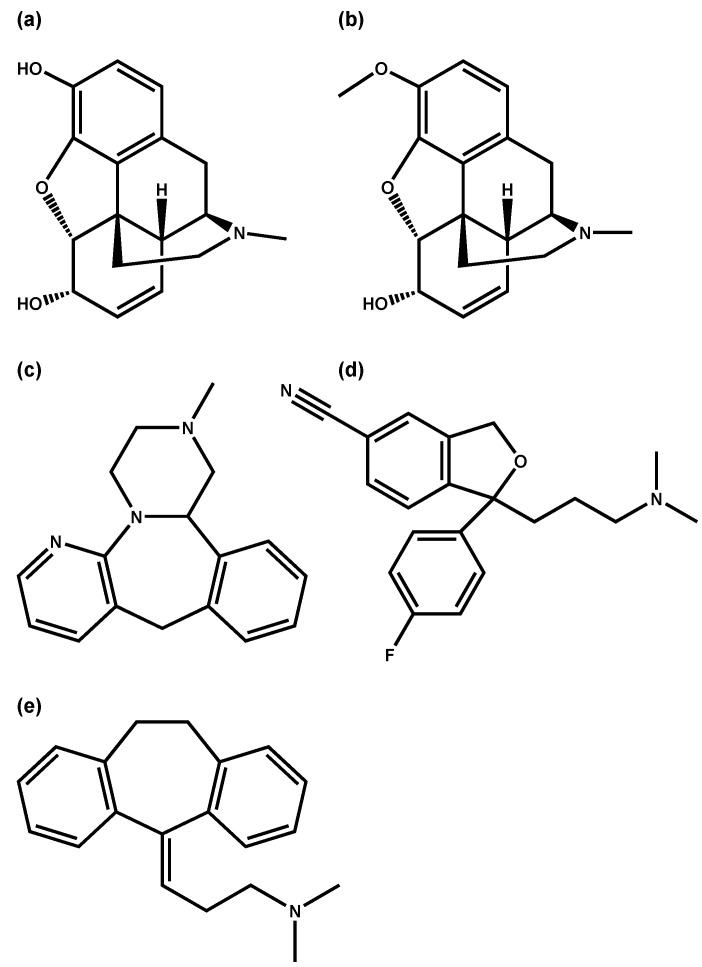
Chemical structures of (**a**) morphine, (**b**) codeine, (**c**) mirtazapine, (**d**) citalopram and (**e**) amitriptyline.

**Figure 4:**
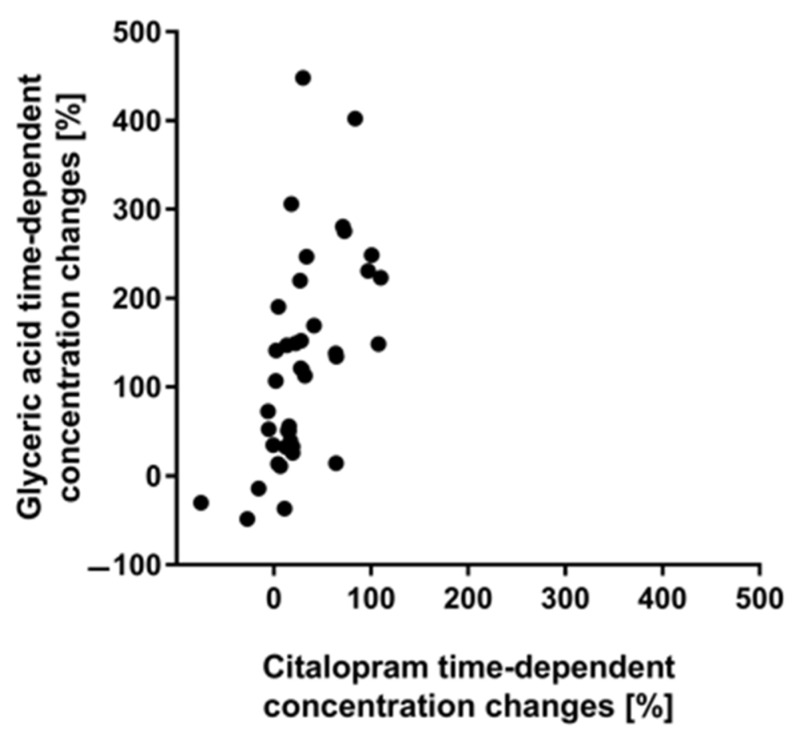
Graphical representation of the correlation analysis; correlation between citalopram and glyceric acid time-dependent concentration changes [%]; x- and y- axis were cut at 500% for better visualization.

As displayed in Table 3, methionine, phenylalanine and valine are among the top four endogenous correlates for morphine, codeine and mirtazapine, but none of the other three studied drugs (diazepam, nordiazepam and citalopram). For morphine and mirtazapine, the correlations with the three essential amino acids were classified as being significant, for codeine only correlation coefficients between 0.42 and 0.45 were observed. Both methionine and valine, along with serine and leucine, were also previously proposed by Langford and Pounder to be potential markers for postmortem drug diffusion from the lung, based on a case study with amitriptyline [16]. Interestingly, morphine, codeine, mirtazapine and also amitriptyline are all chemically comprised of a similar, but not identical, three-ring structure (see Figure 3) which is not found within the chemical structure of citalopram. Based on this, one would expect a very similar time-dependent postmortem behavior of the drugs; however, as detailed above, while morphine and codeine overall do not show significant concentration changes over time, mirtazapine does show an overall trend towards time-dependent concentration increases, similar to citalopram. One could still try to explain the similar correlation pattern of morphine, codeine, mirtazapine and amitriptyline with this structural resemblance. Potential causes for time-dependent changes of the three proteinogenic amino acids are the postmortem degradation of proteins and the chemical degradation of metencephalin, a naturally occurring endogenous opioid peptide that binds to the opioid receptors. Metencephalin is chemically comprised of tyrosine, glycine, phenylalanine and methionine [31]. Furthermore, methionine, phenylalanine and valine exhibiting passive diffusion processes as previously proposed for opioids and antidepressants could explain a correlating postmortem behavior with morphine, codeine and mirtazapine. The best correlation within the current study was found between citalopram and glyceric acid (visualized in Figure 4). The graphical representation shows that while both analytes show time-dependent concentration increases, the extent of concentration changes over time does seem to differ significantly. Generally, time-dependent concentration increases could potentially be caused by passive diffusion processes.

## 3. Materials and Methods

### 3.1. Chemical and Reagents

Methanolic solutions of diazepam, nordiazepam, morphine, codeine, mirtazapine and citalopram (1 mg/mL) and the deuterated internal standards (IS) diazepam-d5, nordiazepam-d5, morphine-d3, codeine-d3, mirtazapine-d3, citalopram-d6 and trimipramine-d3 (0.1 mg/mL) were obtained from Cerilliant (delivered by Sigma-Aldrich, Buchs, Switzerland). Adipic acid, alanine, asparagine, aspartic acid, azelaic acid, cadaverine, caffeine, creatinine, cysteamine, decanoic acid (C10:0), dodecanoic acid (C12:0), eicosanoic acid (C20:0), fructose, galactose, glucose, glutamic acid, glutaric acid, glyceric acid, glycine, heptadecanoic acid (C17:0), hexadecanoic acid (C16:0), hypoxanthine, isoleucine, lactic acid, leucine, lignoceric acid (C24:0), linoleic acid (C18:2), lysine, malic acid, mannose, methionine, niacinamide, nicotinic acid, octadecanoic acid (C18:0), oleic acid (C18:1), ornithine, palmitoleic acid (C16:1), pentadecanoic acid (C15:0), phenylalanine, pipecolic acid, proline, pyroglutamic acid, raffinose, ribose, squalene, suberic acid, succinic acid, tetradecanoic acid (C14:0), threonine, tocopherol, tridecanoic acid (C13:0), tryptamine, tyrosine, uracil, uridine and valine as well as methoxyamine hydrochloride and bovine serum albumin (BSA) in powdered form were purchased from Sigma-Aldrich (Buchs, Switzerland). ISs hippuric acid-15N and testosterone-d2 were obtained from Cambridge Isotope Laboratories, Inc. (Andover, MA, USA). Solutions (1 mL) of N-methyl-N-trimethylsilyl trifluoro acetamide (MSTFA) were sourced from Macherey-Nagel (Düren, Germany). Water was purified with a Purelab Ultra Millipore filtration unit (Labtech, Villmergen, Switzerland). All other chemicals used were of the highest grade available and obtained from Merck (Zug, Switzerland).

### 3.2. Postmortem Sample Collection

Femoral blood samples of 477 cases were collected at two time-points after death (t1 and t2) during routine toxicological investigation at the VIFM (Melbourne, Australia; with ethical approval from the VIFM ethics committee EC20-2019). Upon mortuary admission of a deceased, approximately 2 to 5 mL of postmortem femoral blood was collected by leg puncture as soon as practicable, as per provisions of the Coroners Act 2008 (Victoria). During the medico-legal autopsy, a second femoral blood sample was collected. All postmortem blood samples were preserved in 1% *w*/*v* sodium fluoride and potassium oxalate and stored at 4 °C until shipment. Samples were shipped to the ZIFM (exempt specimens, no import/export permission required) in a temperature-controlled environment at 4 °C and immediately frozen at −80 °C upon receipt until analysis. Anonymized information on estimated time of death, and sampling time-points were provided for further data analysis. Case selection was based on the detectability of one or more drugs (of abuse) independently of the cause of death.

### 3.3. Liquid Chromatography-Tandem Mass Spectrometric Analysis of Drugs (of Abuse)

Sample preparation and targeted quantitative analysis for drugs (of abuse) were carried out with a previously validated method according to Staeheli et al. (83 analytes in 11 postmortem matrices including diazepam, nordiazepam, morphine, codeine, mirtazapine and citalopram; details regarding lower limits of quantification and corresponding calibration ranges see Table S3 in the supplementary material; calibration in whole blood) [19]. In brief, a two-step liquid-liquid extraction (LLE) was used to extract 20 µL femoral blood (butyl acetate/ethyl acetate (1:1, *v*/*v*) at pH 7.4 and pH 13.5). The extracts were combined, evaporated to dryness (N2) and reconstituted in mobile phase (60 µL; eluent A:B (90:10, *v*/*v*); eluent A: 10 mM ammonium formate buffer in water with 0.1% (*v*/*v*) formic acid; eluent B: acetonitrile with 0.1% (*v*/*v*) formic acid). The analysis was conducted on a Thermo Fischer Ultimate 3000 UHPLC system (Thermo Fischer, San Jose, CA, USA) coupled to a Sciex 5500 QTrap linear ion trap quadrupole mass spectrometer (Sciex, Darmstadt, Germany) with instrument settings according to Staeheli et al. [19]. Samples from the same case were prepared and analyzed within the same batch.

### 3.4. Gas Chromatography-High Resolution Mass Spectrometric Analysis of Endogenous Compounds

Extraction and untargeted analysis for endogenous molecules was conducted using a method published by Brockbals et al. (evaluated for 56 endogenous compounds in postmortem femoral blood) [32]. In summary, 20 µL femoral blood was extracted with cooled methanol and a two-step derivatization procedure was carried out (batch-wise methoxymation and on-line silylation). For full scan analysis, a TRACE 1300 GC system (Thermo Scientific, Bremen, Germany) was used, coupled to a Q Exactive GC Orbitrap mass spectrometer (Thermo Scienific, Bremen, Germany) with instrument settings according to Brockbals et al. [32]. Samples were prepared in batches of 13 cases (t1 and t2 of each case were within the same batch) and analyzed in batch-randomized order, with a quality control (QC) sample as every 10th sample (pool of 15 authentic postmortem samples, aliquoted to 80 µL each prior to storage at −20 °C). For semi-quantification purposes, calibration mixes with 56 endogenous compounds at 10 different levels were prepared in an artificial matrix (revised simulated body fluid with addition of 5% (*w*/*w*) bovine serum albumin) as detailed by Brockbals et al. [32].

### 3.5. Data Processing and Data Analysis

#### 3.5.1. Drugs (of Abuse)

IS-corrected quantification of targeted LC-MS/MS data was conducted using MultiQuant^®^ (Version 2.1.1, Sciex, Darmstadt, Germany). For cases where time of death could only be narrowed down to a specific day, but no exact time (*n* = 163), time of death (t0) was defined as 12 pm at the estimated day of death if cases were admitted to the institute on a later day. In the event that admission to the institute was on the same day as the estimated day of death, t0 was specified to be in the middle between 12 am and mortuary admission of the body (t1). These timings were used to calculate the pre-admission and pre-autopsy intervals per case (defined as time between death (t0) and sample collection at mortuary admission (t1)/autopsy (t2)) and are visualized throughout the manuscript. 

#### 3.5.2. Endogenous Compounds

In a first step, GC-HRMS data was processed in a targeted, semi-quantification manner with the TraceFinder™ software (Version 4.1, Thermo Scientific, Bremen, Germany) using a method-specific in-house compound database (details see in the method-specific publication [32]); testoserone-d3 was used for IS-correction for all 56 endogenous molecules. Secondly, GC-HRMS data was also processed with an untargeted metabolomics workflow using Compound Discoverer™ (Version 3.2, Thermo Scientific, Bremen, Germany). Within drug-specific batches, data underwent peak detection, deconvolution, retention time alignment, library matching (orbitrap GC-MS HRAM metabolomics library, Thermo Scientific, Bremen, Germany) and normalization (constant median). A summary of processing parameters is listed in Table 4. Based on drug/endogenous concentrations and normalized peak areas, the percentage concentration changes between t1 and t2 were calculated ((t21)/t1*100) for each drug/endogenous molecule/feature per case. 

#### 3.5.3. Mixed Effect Models

Using the drugs (of abuse) quantification results for t1 and t2, a mixed effect model per drug (of abuse) was created according to the following formula with ∆t being the time-interval between t1 and t2 (t1 − t2) using R [33]:

c(t1) = c(t2) ∗ e^(λ(∆t)).(3)

The following R packages were utilized: data.table [34], gridExtra [35], lme4 [36], lubridate [37], outliers [38] and tidyverse [39]. In a first step, randomly selected 80% of the cases were used for the creation of the model (training set) with the remaining 20% of the cases being used as a test set. To check for robustness and reproducibility of the models, model creation was conducted 50 times and RSD of the lambda (λ) values were calculated. Accuracy values were calculated comparing back-calculated t1 concentrations with actual quantification data for t1. For all models, the 2.5 to 97.5 % confidence intervals were calculated. 

#### 3.5.4. Correlation Analysis

Spearman rank correlation analysis was carried out to establish correlations between the percentage concentration changes of a drug (diazepam/nordiazepam/morphine/codeine/mirtazapine/citalopram) and time-dependent concentration changes of targeted endogenous molecules/features using MetaboAnalyst. Significance of the obtained correlation coefficients (*p*-value) were corrected for multiple testing using FDR. For the current study, spearman rank correlation coefficients of greater than 0.5 and smaller than −0.5 with a *p*-value (FDR-corrected) of smaller than 0.05 were defined as significant.

## 4. Conclusions

The current study comprises one of the most extensive data sets in the context of time-dependent postmortem studies. This allowed for the first-time mathematical modelling of the time-dependent postmortem behavior of diazepam and nordiazepam with the view to estimate the drug concentrations closer to the estimated time of death. While it might give a general indication on the extent of diazepam/nordiazepam time-dependent postmortem concentration decreases, particularly for cases with an extensive postmortem interval until sampling, inter-individual variations make the transfer of these models to case specific forensic interpretation difficult. Additionally, the current concept that endogenous molecules could parallel drug redistribution mechanisms could not be confirmed. The idea was to search for endogenous correlates for easy, targeted routine use for an *a posteriori* prediction of PMR occurrence, which could also be used as surrogates to further understand the underlying processes of PMR. However, very strong correlations between one of the analyzed drugs (of abuse) and an endogenous molecule/feature were not observed within the current GC-HRMS study. Complementing the current results with a LC-MS/MS metabolomics study could lead to an increased number of significant endogenous correlates. Additionally, incorporating the amino acids methionine, phenylalanine and valine into current targeted routine postmortem methods might help to further understand their time-dependent postmortem behavior and to define meaningful postmortem concentration ranges that might help in further PMR studies. Nevertheless, the current dataset did successfully lead to a significant knowledge gain in further understanding the time-dependent postmortem behavior of diazepam, nordiazepam, morphine, codeine, mirtazapine, and citalopram that can be useful for forensic toxicological case interpretation. For the future, additional concepts to deepen the understanding of PMR processes need to be developed.

## Figures and Tables

**Table 1:** Summary of time-intervals between estimated time of death and sampling time-points.

-	Pre-Admission interval (t0 − t1) [h]	Pre-Autopsy Interval (t0 − t2) [h]	∆t (t1 − t2) [h]
**Mean**	16	99	83
**Median**	9	86	70
**Min**	1.25	11	6.5
**Max**	292	478	434

**Table 2:** Summary data on the parameters for the mixed effect models; RSD refers to the relative standard deviation of lambda upon repeating model creation with a randomly selected training set for 50 times.

-	Diazepam	Nordiazepam	Morphine	Codeine	Mirtazapine	Citalopram
Lambda (λ)	−0.00296044	−0.00303677	0.0014037	0.00020796	0.00333899	0.0037752
RSD of λ [%]	7.2	5.4			27	15
Confidence interval (2.5–97.5%)	−0.00352471 to −0.00239861	−0.00365846 to −0.00241503	−0.00023068 to 0.00302557	−0.00078685 to 0.00119634	0.00035995 to 0.00627887	0.00139422 to 0.00619429
Median prediction accuracy [%]	109	108				

**Table 3:** Endogenous compounds/features that were found to have the highest spearman correlation coefficients with the corresponding drugs (of abuse); *p*-values are corrected for multiple testing using false discovery rate (FDR); it is indicated with which workflow the endogenous compound was found and the identification level according to the chemical analysis working group (CAWG) of the metabolomics standards initiative (MSI) [30].

Drug	Endogenous Compound/Feature	Spearman Correlation Coefficient	*p*-Value (FDR)	Workflow/ID Level
Diazepam *n* = 137	Fumaric acid 2TMS	0.38394	<0.05	untargeted/2
	Hexadecanoic acid (C16:0) TMS	0.36859	<0.05	targeted/1
	Oleic acid (C18:1) TMS	0.35069	<0.05	untargeted/1
	Glyceric acid 3TMS	−0.38241	<0.05	untargeted/1
Nordiazepam *n* = 126	Fumaric acid 2TMS	0.33849	<0.05	untargeted/2
	Ornithine 4TMS	0.32831	<0.05	untargeted/1
	Oleic acid (C18:1) TMS	0.35069	<0.05	untargeted/1
	Glyceric acid 3TMS	−0.46592	<0.05	targeted/1
	10.47_219.12279	−0.32552	<0.05	untargeted/4
Morphine *n* = 122	Methionine 2TMS	0.57252	<0.05	targeted/1
	Phenylalanine 2TMS	0.55928	<0.05	targeted/1
	Valine 2TMS	0.56490	<0.05	targeted/1
	Isoleucine 2TMS	0.52284	<0.05	targeted/1
	Proline 2TMS	0.51051	<0.05	targeted/1
Codeine *n* = 92	Methionine 2TMS	0.45389	<0.05	targeted/1
	Oleic acid (C18:1) TMS	0.45302	<0.05	targeted/1
	Phenylalanine 2TMS	0.44358	<0.05	targeted/1
	Valine 2TMS	0.42247	<0.05	targeted/1
	11.679_245.10248	−0.41774	<0.05	untargeted/4
Mirtazapine *n* = 55	Methionine 2TMS	0.58152	<0.05	targeted/1
	Uracil 2TMS	0.57225	<0.05	targeted/1
	Phenylalanine 2TMS	0.56394	<0.05	targeted/1
	Valine 2TMS	0.51234	<0.05	targeted/1
Citalopram *n* = 50	Glyceric acid 3TMS	0.60497	<0.05	targeted/1
	Ribose 4TMS 1MOX	0.55732	<0.05	targeted/1
	Alanine 2TMS	0.52508	<0.05	targeted/1

**Table 4:** Summary of processing parameters within the “Search Spectrum” and “GC EI deconvolution” node of the Compound Discoverer™ software (Version 3.2, Thermo Scientific, Bremen, Germany); S/N: signal-to-noise-ratio; TIC: total ion chromatogram.

**Spectrum Properties Filter**	
Lower retention time limit	5.3 min
Upper retention time limit	24.5 min
**Peak Detection Settings**	
Mass tolerance	5 ppm
Spectral S/N threshold	3
Peak S/N threshold	5
Smoothing	9
TIC threshold	500,000
Ion overlap window	98%
**Group Compounds Settings**	
Retention time tolerance	10 s
Dot product threshold	500
Composition threshold	10

## Data Availability

The data presented in this study are available on request from the corresponding author. The data are not publicly available due to privacy and ethical reasons.

## Supplementary Materials

The following are available online at www.mdpi.com/article/10.3390/metabo11090643/s1, Figure S1: Direct visual comparison of the quantification results of the Victorian Institute of Forensic Medicine (VIFM; x-axis; in ng/mL) against the Zurich Institute of Forensic Medicine (ZIFM; y-axis; in ng/mL), Figure S2: Visual representation of the Bland-Altman analyses; %Difference was calculated as (100x(B-A)/Average) with A being the quantification results of the Zurich Institute of Forensic Medicine (ZIFM) and B being the initial quantification results of the Victorian Institute of Forensic Medicine (VIFM), Table S1: Raw data (drug concentrations) with calculated concentration changes and sampling timings per case, sorted by drug (of abuse); concentration values below the lower limit of quantification are given in italic, Table S2: Individual accuracy values for diazepam and nordiazepam mixed effect models; “mathematically calculated concentrations of t1” were calculated based on t2 analytically measured concentration by taking into account ∆t; accuracies compare t1-measured concentration with t1-mathematically calculated concentration, Table S3: List of lower limits of quantification (LLOQ) for the discussed drugs (of abuse) including their corresponding calibration ranges (12C and 13C calibration).
