# Supplementary material for: Postmortem Metabolomics: Strategies to Assess Time-Dependent Postmortem Changes of Diazepam, Nordiazepam, Morphine, Codeine, Mirtazapine and Citalopram
Source: Metabolites. 2021 Sep 20;11(9):643. doi: 10.3390/metabo11090643 (PMC8466227; doi:10.3390/metabo11090643)
Supplement: Supplementary file 1 [file metabolites-11-00643-s001.zip › metabolites-1366568-supplementary.pdf]

Supplementary Materials

Postmortem metabolomics: Strategies to assess time-dependent postmortem changes of diazepam, nordiazepam, morphine, codeine, mirtazapine and citalopram

Lana Brockbals [1], Yannick Wartmann [1], Dylan Mantinieks [2,3], Linda L. Glowacki [3], Dimitri Gerostamoulos [2,3] Thomas Kraemer [1] and Andrea E. Steuer [1,*]

[1] Department of Forensic Pharmacology and Toxicology, Zurich Institute of Forensic Medicine, University of Zurich, Winterthurerstrasse 190/52, 8057 Zurich, Switzerland
[2] Department of Forensic Medicine, Monash University, 65 Kavanagh Street, Southbank 3006, Victoria, Australia
[3] Victorian Institute of Forensic Medicine, 65 Kavanagh Street, Southbank 3006, Victoria, Australia
* Correspondence: andrea.steuer@irm.uzh.ch

Figure S1: Direct visual comparison of the quantification results of the Victorian Institute of Forensic Medicine (VIFM; x-axis; in ng/mL) against the Zurich Institute of Forensic Medicine (ZIFM; y-axis; in ng/mL); straight line in all graphs follows the formula y = x, representing the perfect agreement between VIFM and ZIFM quantification data.

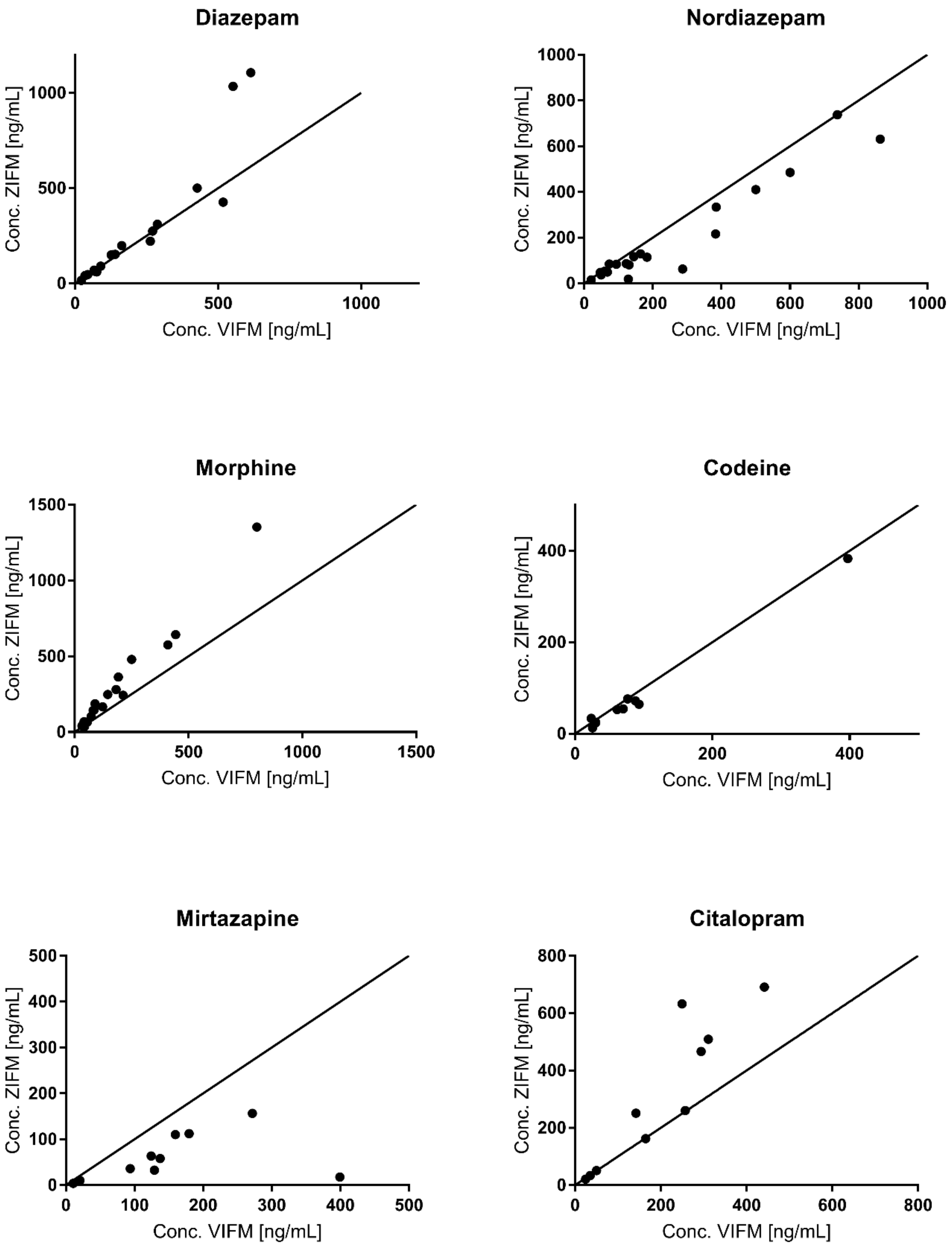

Figure S2: Visual representation of the Bland-Altman analyses; %Difference was calculated as (100x(B-A)/Average) with A being the quantification results of the Zurich Institute of Forensic Medicine (ZIFM) and B being the initial quantification results of the Victorian Institute of Forensic Medicine (VIFM).

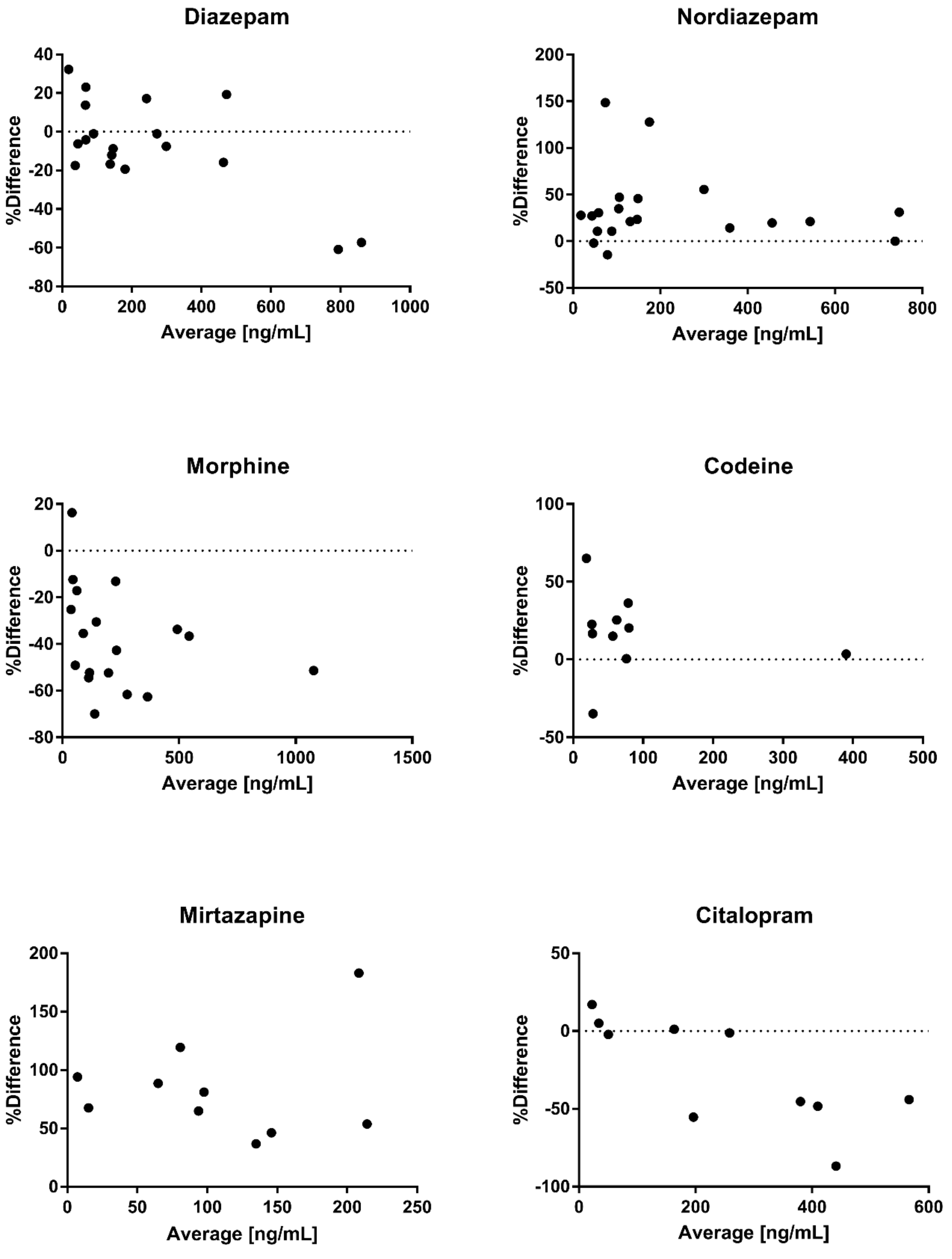

Table S1: Raw data (drug concentrations) with calculated concentration changes and sampling timings per case, sorted by drug (of abuse); concentration values below the lower limit of quantification are given in italic.

Drug (of abuse)	Case number	Concentration [ng/mL]		Time-dependent concentration change [%]	Pre-admission interval (t0-t1) [h]	Pre-autopsy interval (t0-t2) [h]	Δt (t1-t2) [h]
		t1	t2				
Diazepam	2308	30	18	-41	3.0	123	120
	2330	29	24	-16	8.9	244	235
	2339	41	26	-36	6.8	77	70
	2371	4.5	14	218	22	141	119
	2388	19	8.9	-54	4.8	159	155
	2432	11	7.4	-32	16	131	115
	2438	62	32	-49	7.9	221	213
	2451	150	96	-37	3.2	61	57
	2498	340	240	-30	3.5	139	136
	2525	78	110	43	8.6	77	68
	2561	140	70	-51	5.4	16	11
	2570	440	210	-52	4.2	198	194
	2590	110	54	-52	2.8	144	141
	2612	17	14	-18	18	270	252
	2622	13	25	87	1.8	72	70
	2651	430	580	36	5.7	29	23
	2656	5.7	*5.0*	-13	8.0	150	142
	2658	60	19	-68	8.6	29	20
	2665	270	220	-20	14	25	12
	2677	200	65	-68	19	163	144
	2688	87	54	-38	21	67	46
	2711	310	210	-32	6.3	27	21
	2752	500	140	-72	8.9	123	114
	2754	460	470	3.1	6.0	47	41
	2771	260	160	-40	12	93	80
	2772	36	35	-2.0	14	120	106
	2780	26	14	-45	6.3	97	90
	2788	430	470	8.6	15	38	22
	2789	71	53	-26	16	36	21
	2800	200	160	-24	3.1	50	46
	2811	95	68	-29	25	91	66
	2816	470	310	-35	15	154	139
	2817	310	150	-52	7.3	53	46
	2838	59	36	-39	127	197	70
	2839	10	11	10	28	120	92
	2843	300	360	21	4.8	15	10
	2876	39	31	-21	1.8	57	55
	2878	49	37	-25	26	97	71
	2895	85	82	-4.0	5.9	50	44
	2918	15	6.1	-58	5.0	76	71
	2924	10	10	-0.5	11	26	15
	2927	57	130	132	11	145	134
	2970	70	49	-29	3.4	91	88

	2978	31	24	-23	5.1	98	93
	2979	91	51	-43	6.1	76	70
	2999	80	81	1.4	3.4	15	12
	3031	260	230	-12	2.2	31	29
	3033	11	*4.5*	-59	6.7	173	167
	3037	7.5	*2.9*	-61	78	192	114
	3044	37	25	-32	23	114	91
	3060	16	11	-27	4.4	120	116
	3073	220	180	-17	9.7	46	36
	3079	210	160	-25	5.1	38	33
	3080	280	89	-69	4.0	300	296
	3085	30	14	-52	6.9	76	69
	3089	410	290	-30	8.2	127	119
	3115	89	56	-37	6.9	188	181
	3121	*4.2*	5.5	28	32	192	160
	3130	250	270	7.2	10	61	51
	3131	100	92	-11	3.8	28	24
	3137	130	130	6.0	4.6	42	38
	3168	84	120	37	5.0	66	61
	3174	110	46	-57	2.8	154	151
	3180	100	85	-15	2.7	383	381
	3200	82	51	-38	11	72	62
	3205	130	110	-11	5.9	85	79
	3209	75	83	10	2.4	31	28
	3227	120	67	-42	9.3	147	138
	3243	42	38	-9.2	9.4	27	17
	3249	62	58	-5.9	6.6	89	82
	3312	1100	670	-40	4.0	28	24
	3315	24	27	14	11	27	17
	3324	150	120	-21	28	119	91
	3328	10	6.3	-37	13	119	106
	3351	47	46	-2.6	4.0	59	55
	3368	14	16	8.3	3.6	28	24
	3375	48	24	-50	34	143	109
	3380	240	190	-21	12	47	36
	3442	21	17	-21	8.6	99	90
	3443	26	14	-48	9.9	75	65
	3448	200	68	-66	14	172	158
	3459	22	23	4.3	2.0	34	32
	3467	62	31	-50	9.9	338	328
	3483	27	42	52	3.1	98	94
	3489	140	54	-61	5.4	115	110
	3495	7.6	5.2	-31	5.7	113	107
	3505	52	45	-14	20	142	122
	3518	170	80	-51	4.0	85	81
	3519	110	120	7.9	31	51	20
	3524	150	80	-46	5.4	51	46
	3537	19	12	-39	8.0	82	74
	3541	50	35	-29	6.6	74	67
	3547	66	64	-3.2	6.2	46	39
	3554	16	8.4	-47	25	170	145

	3568	360	290	-19	49	144	95
	3573	15	31	101	14	25	11
	3580	110	86	-24	3.8	27	24
	3591	19	18	-6.0	21	87	67
	3595	120	65	-45	1.9	106	104
	3617	41	23	-43	6.3	77	71
	3633	110	82	-22	98	191	94
	3637	110	53	-50	51	121	70
	3638	47	22	-52	1.9	40	39
	3652	79	35	-56	9.8	138	128
	3654	24	11	-52	15	153	138
	3660	47	29	-38	7.2	148	141
	3674	69	35	-50	31	143	112
	3676	64	48	-25	87	222	135
	3692	110	90	-19	4.3	107	103
	3715	150	160	7.0	3.5	143	140
	3721	6.9	7.1	3.0	3.5	14	11
	3724	18	15	-18	6.5	54	48
	3753	41	26	-36	11	72	62
	3760	250	220	-14	7.4	199	192
	3778	53	28	-47	4.5	219	214
	3787	*4.9*	*4.8*	-1.7	8.1	144	136
	3821	210	290	36	3.0	50	47
	3824	44	39	-11	2.8	19	16
	3834	290	170	-43	12	143	132
	3849	390	410	3.7	11	49	38
	3859	53	61	14	4.0	141	137
	3861	6.8	*3.9*	-43	9.7	146	136
	3878	62	34	-45	12	144	133
	3888	200	130	-34	3.0	118	115
	3890	40	40	-0.2	13	37	24
	3891	34	22	-35	54	75	21
	3900	240	220	-9.0	2.0	66	64
	3932	140	96	-31	3.8	148	144
	3948	46	47	1.6	16	114	98
	3954	33	22	-34	4.2	125	121
	3960	280	300	6.2	4.4	89	84
	3985	34	32	-5.3	4.5	77	72
	3995	1000	1500	48	12	22	10
	4006	58	42	-28	8.3	57	49
	4015	150	97	-34	3.9	119	115
	4029	390	270	-32	8.4	29	20
	4075	210	150	-30	9.1	53	44
Nordiazepam	2330	41	31	-26	8.9	244	235
	2339	26	18	-33	6.8	77	70
	2371	17	25	53	22	141	119
	2438	35	*5.5*	-84	7.9	221	213
	2451	50	32	-35	3.2	61	57
	2483	20	8.4	-58	4.8	78	73
	2498	350	250	-30	3.5	139	136
	2525	94	130	36	8.6	77	68

	2526	71	100	42	8.3	151	143
	2561	180	87	-52	5.4	16	11
	2570	300	150	-51	4.2	198	194
	2581	15	14	-4.3	4.1	38	34
	2590	93	39	-58	2.8	144	141
	2622	78	130	69	1.8	72	70
	2651	410	430	4.3	5.7	29	23
	2658	63	48	-24	8.6	29	20
	2665	86	75	-13	14	25	12
	2677	170	130	-22	19	163	144
	2688	140	91	-35	21	67	46
	2697	14	*6.7*	-51	5.2	216	210
	2711	120	76	-35	6.3	27	21
	2752	740	310	-58	8.9	123	114
	2754	580	560	-4.0	6.0	47	41
	2771	320	180	-42	12	93	80
	2772	93	79	-15	14	120	106
	2788	62	63	0.4	15	38	22
	2789	31	21	-30	16	36	21
	2800	170	140	-13	3.1	50	46
	2811	43	27	-38	25	91	66
	2816	450	310	-31	15	154	139
	2817	280	110	-62	7.3	53	46
	2838	39	20	-49	127	197	70
	2843	29	37	26	4.8	15	10
	2876	14	12	-10	1.8	57	55
	2878	190	160	-16	26	97	71
	2895	34	25	-25	5.9	50	44
	2918	67	35	-48	5.0	76	71
	2927	50	50	0.2	11	145	134
	2970	99	77	-22	3.4	91	88
	2978	23	17	-24	5.1	98	93
	2979	330	230	-32	6.1	76	70
	2999	240	230	-5.3	3.4	15	12
	3031	170	150	-12	2.2	31	29
	3033	19	25	31	6.7	173	167
	3044	17	*9.2*	-46	23	114	91
	3073	120	97	-15	9.7	46	36
	3079	210	170	-22	5.1	38	33
	3080	340	120	-66	4.0	300	296
	3085	12	*5.4*	-55	6.9	76	69
	3089	130	88	-33	8.2	127	119
	3115	110	85	-26	6.9	188	181
	3130	150	130	-13	10	61	51
	3131	110	100	-9.1	3.8	28	24
	3137	69	65	-5.4	4.6	42	38
	3168	84	110	25	5.0	66	61
	3174	39	17	-57	2.8	154	151
	3180	400	290	-27	2.7	383	381
	3200	240	150	-37	11	72	62
	3205	420	360	-13	5.9	85	79

	3209	68	47	-30	2.4	31	28
	3227	56	33	-41	9.3	147	138
	3243	33	32	-1.1	9.4	27	17
	3249	110	82	-27	6.6	89	82
	3312	630	410	-35	4.0	28	24
	3315	16	21	29	11	27	17
	3324	220	180	-16	28	119	91
	3351	29	25	-12	4.0	59	55
	3375	14	4.3	-68	34	143	109
	3380	39	32	-17	12	47	36
	3442	47	27	-42	8.6	99	90
	3448	490	180	-63	14	172	158
	3459	50	55	8.7	2.0	34	32
	3467	81	44	-45	9.9	338	328
	3483	39	51	29	3.1	98	94
	3489	350	120	-67	5.4	115	110
	3495	26	13	-49	5.7	113	107
	3505	93	77	-18	20	142	122
	3518	30	12	-60	4.0	85	81
	3519	190	2100	5.7	31	51	20
	3524	48	24	-50	5.4	51	46
	3537	84	45	-46	8.0	82	74
	3541	160	100	-36	6.6	74	67
	3547	270	250	-9.0	6.2	46	39
	3554	17	*7.1*	-59	25	170	145
	3565	18	16	-12	24	121	97
	3568	250	240	-4.9	49	144	95
	3573	120	120	4.2	14	25	11
	3580	180	130	-29	3.8	27	24
	3591	120	100	-12	21	87	67
	3595	270	160	-39	1.9	106	104
	3610	20	20	3.2	60	95	35
	3617	52	29	-44	6.3	77	71
	3633	48	50	2.9	98	191	94
	3637	77	55	-28	51	121	70
	3638	44	22	-51	1.9	40	39
	3652	120	44	-62	9.8	138	128
	3660	130	100	-22	7.2	148	141
	3674	37	21	-44	31	143	112
	3676	84	75	-10	87	222	135
	3692	37	25	-32	4.3	107	103
	3715	270	260	-5.7	3.5	143	140
	3721	17	17	2.2	3.5	14	11
	3753	84	52	-38	11	72	62
	3760	200	160	-20	7.4	199	192
	3778	140	91	-36	4.5	219	214
	3787	15	11	-31	8.1	144	136
	3821	74	78	5.1	3.0	50	47
	3824	66	57	-13	2.8	19	16
	3834	310	190	-37	12	143	132
	3849	240	170	-28	11	49	38

	3859	77	47	-38	4.0	141	137
	3878	57	34	-41	12	144	133
	3888	170	95	-43	3.0	118	115
	3890	84	84	0.4	13	37	24
	3900	170	150	-14	2.0	66	64
	3906	13	*8.7*	-32	20	114	95
	3932	180	110	-36	3.8	148	144
	3948	37	26	-28	16	114	98
	3954	120	70	-40	4.2	125	121
	3960	75	74	-2.1	4.4	89	84
	3985	49	36	-28	4.5	77	72
	3995	53	73	38	12	22	10
	4006	65	55	-16	8.3	57	49
	4015	640	320	-50	3.9	119	115
	4029	210	150	-30	8.4	29	20
	4075	230	240	3.9	9.1	53	44
Morphine	2371	490	8.1	-98	22	141	119
	2392	300	660	121	14	183	169
	2438	140	6.7	-95	7.9	221	213
	2451	41	27	-34	3.2	61	57
	2479	18	13	-30	16	199	183
	2531	29	79	172	30	83	53
	2582	65	76	17	18	46	28
	2597	150	250	65	5.2	103	98
	2598	3.2	5.8	84	5.9	74	68
	2614	57	63	10	16	149	133
	2622	510	690	36	1.8	72	70
	2626	2.1	4.0	87	8.9	114	105
	2656	30	65	116	8.0	150	142
	2723	34	35	3.8	11	25	14
	2739	34	41	21	21	51	31
	2771	290	250	-14	12	93	80
	2780	19	21	13	6.3	97	90
	2793	8.0	140	1615	23	70	47
	2799	41	48	15	51	72	21
	2838	140	110	-18	127	197	70
	2843	19	20	1.7	4.8	15	10
	2878	48	51	6.5	26	97	71
	2885	68	98	45	149	212	64
	2904	72	58	-20	40	79	39
	2908	220	230	3.8	18	40	22
	2918	670	530	-21	5.0	76	71
	2923	120	120	6.5	22	48	26
	2926	47	75	59	4.0	140	136
	2927	42	*0.2*	-100	11	145	134
	2941	180	240	39	15	43	28
	2942	41	42	2.4	1.5	37	36
	2962	83	200	136	9.8	126	117
	2970	410	380	-8.4	3.4	91	88
	2973	50	48	-3.2	22	145	122
	2978	140	150	8.1	5.1	98	93

	2979	37	370	916	6.1	76	70
	2985	18	17	-2.6	19	68	49
	2999	370	330	-11	3.4	15	12
	3007	75	86	15	3.8	63	59
	3012	4.1	4.8	19	20	47	26
	3020	150	5.8	-96	31	48	17
	3031	190	200	8.2	2.2	31	29
	3040	16	420	2462	18	139	121
	3043	150	62	-59	11	49	39
	3044	100	88	-12	23	114	91
	3056	290	310	9.0	3.1	133	130
	3058	28	31	10	6.1	152	145
	3067	190	75	-61	3.3	56	52
	3100	50	45	-10	13	83	71
	3107	7.9	12	49	33	199	166
	3137	1400	1500	8.8	4.6	42	38
	3142	67	68	2.5	4.8	157	153
	3155	1400	1600	21	1.3	130	129
	3168	72	87	20	5.0	66	61
	3174	41	86	109	2.8	154	151
	3180	170	220	32	2.7	383	381
	3188	9.2	650	6979	17	116	99
	3232	160	160	-1.8	14	21	6
	3277	250	340	35	12	191	179
	3288	110	67	-36	23	96	72
	3299	150	120	-21	5.8	56	50
	3302	140	120	-15	72	97	24
	3307	150	140	-5.8	18	38	21
	3320	96	74	-22	194	241	47
	3346	72	78	8.7	27	280	253
	3351	250	240	-2.7	4.0	59	55
	3380	350	330	-5.0	12	47	36
	3382	49	130	163	15	73	58
	3401	180	200	13	18	34	16
	3403	4.5	4.1	-9.0	16	124	108
	3442	600	270	-56	8.6	99	90
	3448	190	190	3.8	14	172	158
	3489	6.1	5.8	-4.4	5.4	115	110
	3495	33	30	-8.1	5.7	113	107
	3505	11	41	262	20	142	122
	3517	29	24	-18	5.2	61	56
	3521	140	170	26	13	77	64
	3537	140	190	36	8.0	82	74
	3547	650	800	23	6.2	46	39
	3555	68	67	-0.4	37	56	19
	3577	220	250	11	4.2	101	97
	3587	580	570	-1.0	9.0	71	62
	3595	8.6	31	257	1.9	106	104
	3605	4.0	19	384	23	163	139
	3656	92	83	-9.4	7.7	126	118
	3674	4.0	5.0	26	31	143	112

	3685	180	690	290	12	121	109
	3692	110	130	18	4.3	107	103
	3724	150	140	-1.0	6.5	54	48
	3748	280	300	5.0	11	169	159
	3753	360	300	-17	11	72	62
	3760	8.3	9.7	18	7.4	199	192
	3778	340	870	159	4.5	219	214
	3787	17	49	181	8.1	144	136
	3793	310	280	-10	4.0	278	274
	3830	110	110	0.0	3.3	99	95
	3848	99	100	2.4	11	21	10
	3849	330	150	-54	11	49	38
	3859	16	2.6	-83	4.0	141	137
	3861	23	170	663	9.7	146	136
	3888	50	50	0.8	3.0	118	115
	3891	71	86	21	54	75	21
	3900	980	860	-12	2.0	66	64
	3904	36	52	46	3.7	206	203
	3932	74	130	71	3.8	148	144
	3944	95	77	-19	50	111	61
	3948	5.6	12	115	16	114	98
	3953	4.1	23	446	6.3	124	118
	3963	640	430	-33	4.4	69	64
	3971	79	74	-6.8	23	115	92
	3980	36	55	51	14	38	23
	3986	20	18	-11	5.1	25	19
	3989	180	300	62	2.1	73	71
	4003	170	170	1.5	7.2	148	141
	4006	460	520	13	8.3	57	49
	4008	16	18	19	4.7	31	26
	4015	40	27	-33	3.9	119	115
	4042	160	170	9.3	6.2	77	71
	4045	16	16	-3.3	26	73	47
	4055	93	71	-23	2.1	84	82
	4066	480	410	-15	4.1	43	39
	4071	170	130	-22	5.3	56	51
Codeine	2390	76	100	34	3.5	159	156
	2438	14	8.0	-43	7.9	221	213
	2451	7.6	5.3	-31	3.2	61	57
	2522	2.4	2.6	6.0	11	146	135
	2532	23	33	45	6.5	99	92
	2535	26	30	14	5.6	142	136
	2536	27	30	8.0	8.9	123	114
	2591	28	27	-4.3	8.0	125	117
	2598	60	120	98	5.9	74	68
	2622	32	53	63	1.8	72	70
	2644	17	40	139	7.4	174	166
	2654	34	10	-70	8.1	54	46
	2656	11	18	65	8.0	150	142
	2674	3.8	6.4	70	11	97	86
	2761	55	74	34	142	240	98

	2771	27	25	-10	12	93	80
	2780	3.1	3.1	-0.3	6.3	97	90
	2800	5.1	5.8	15	3.1	50	46
	2816	48	52	9.2	15	154	139
	2842	4.5	20	345	3.0	41	38
	2843	1000	1200	16	4.8	15	10
	2918	2.4	*1.9*	-20	5.0	76	71
	2926	8.7	7.7	-12	4.0	140	136
	2927	6.4	4.5	-29	11	145	134
	2970	26	25	-3.7	3.4	91	88
	2973	64	120	83	23	145	122
	2978	25	24	-1.3	5.1	98	93
	2979	11	20	72	6.1	76	70
	2985	29	33	16	19	68	49
	3007	17	21	24	3.8	63	59
	3020	21	9.2	-55	31	48	17
	3031	28	32	16	2.2	31	29
	3043	12	6.9	-40	11	49	39
	3056	26	25	-2.2	3.1	133	130
	3084	2.2	14	559	6.0	103	97
	3131	4.2	3.9	-6.6	3.8	28	24
	3137	88	110	25	4.6	42	38
	3142	10	8.4	-17	4.8	157	153
	3168	10	12	15	5.0	66	61
	3174	30	36	21	2.8	154	151
	3180	19	21	10	2.7	383	381
	3205	7.8	11	45	5.9	85	79
	3227	61	73	19	9.3	147	138
	3299	32	21	-36	5.8	56	50
	3312	380	350	-9.5	4.0	28	24
	3346	9.8	8.4	-14	27	280	253
	3351	33	30	-10	4.0	59	55
	3366	50	59	18	2.2	40	38
	3380	37	37	-1.8	12	47	36
	3382	19	29	54	15	73	58
	3442	48	21	-56	8.6	99	90
	3443	3.4	3.7	11	9.9	75	65
	3480	24	29	23	3.5	26	22
	3489	320	270	-18	5.4	115	110
	3495	110	89	-17	5.7	113	107
	3524	15	15	-2.7	5.4	51	46
	3547	87	100	18	6.2	46	39
	3554	9.0	7.5	-16	25	170	145
	3577	28	28	-0.9	4.2	101	97
	3656	11	11	0.6	7.7	126	118
	3674	53	50	-5.1	31	143	112
	3685	29	35	20	12	121	109
	3692	17	22	24	4.3	107	103
	3698	7.3	11	55	4.6	141	137
	3724	25	25	-1.5	6.5	54	48
	3748	72	68	-5.6	11	169	159

	3753	76	68	-10	11	72	62
	3760	2.5	2.4	-5.4	7.4	199	192
	3763	3.6	4.1	15	10	145	135
	3787	3.2	3.7	13	8.1	144	136
	3793	52	46	-11	4.0	278	274
	3830	9.5	8.7	-8.5	3.3	99	95
	3849	33	18	-45	11	49	38
	3861	4.6	14	213	9.7	146	136
	3888	6.4	5.8	-9.5	3.0	118	115
	3893	2.7	2.7	-1.9	7.4	100	92
	3900	66	58	-13	2.0	66	64
	3904	6.2	6.7	8.4	3.7	206	203
	3932	13	15	15	3.8	148	144
	3953	24	37	57	6.3	124	118
	3963	65	37	-43	4.4	69	64
	3986	650	630	-3.4	5.1	25	19
	3989	16	29	81	2.1	73	71
	3995	4.4	80	1697	12	22	10
	4003	13	13	4.7	7.2	148	141
	4006	210	280	34	8.3	57	49
	4015	960	710	-26	3.9	119	115
	4042	30	29	-3.5	6.2	77	71
	4055	7.8	5.0	-36	2.1	84	82
	4066	54	48	-11	4.1	43	39
	4071	15	12	-22	5.3	56	51
	4092	41	40	-1.9	11	50	39
Mirtazapine	2322	86	300	254	4.9	132	127
	2372	110	8.4	-92	4.0	22	18
	2438	140	7.0	-95	7.9	221	213
	2535	63	110	69	5.6	142	136
	2581	110	200	80	4.1	38	34
	2614	53	78	49	16	149	133
	2655	*3.5*	7.1	102	8.4	26	18
	2658	18	120	560	8.6	29	20
	2824	130	290	121	27	186	159
	2835	26	12	-53	7.7	28	21
	2838	*3.1*	13	319	127	197	70
	2855	36	130	264	6.3	100	93
	2869	140	290	113	9.4	218	209
	2898	98	170	72	3.4	50	46
	2924	20	59	198	11	26	15
	2955	34	100	200	2.8	87	84
	2970	*3.4*	17	400	3.4	91	88
	2973	290	340	14	23	145	122
	2985	24	32	35	19	68	49
	2999	24	26	7.8	3.4	15	12
	3013	8.0	86	965	28	65	37
	3014	43	5.3	-87	4.3	44	40
	3122	64	120	92	2.8	18	16
	3131	280	370	30	3.8	28	24
	3132	85	210	150	4.1	163	159

	3243	33	48	49	9.4	27	17
	3320	13	11	-19	194	241	47
	3323	38	48	28	10	26	16
	3363	*3.9*	*4.4*	12	8.8	28	19
	3372	98	260	161	4.7	51	47
	3384	110	280	145	5.6	127	121
	3421	50	160	224	9.4	122	113
	3424	10	23	125	11	74	64
	3430	16	30	88	28	76	48
	3440	21	34	66	5.7	73	67
	3451	200	340	72	6.7	101	94
	3483	18	38	109	3.1	98	94
	3513	220	110	-50	48	84	36
	3524	58	67	15	5.4	51	46
	3568	31	60	91	49	144	95
	3622	17	20	21	26	68	43
	3630	33	120	278	8.6	99	91
	3637	49	160	214	51	121	70
	3742	25	44	73	5.2	52	47
	3755	160	310	97	3.4	177	174
	3767	51	77	50	6.8	186	180
	3769	63	120	81	12	192	180
	3812	18	31	73	4.1	196	192
	3878	73	220	203	12	144	133
	3890	44	61	38	13	37	24
	3927	62	140	117	12	69	57
	3932	35	67	92	3.8	148	144
	3944	48	9.3	-81	50	111	61
	4033	51	96	91	11	29	18
	4045	140	200	42	26	73	47
Citalopram	2303	200	200	-0.5	13	24	11
	2308	300	510	73	3.0	123	120
	2542	170	9.3	-95	12	28	16
	2582	250	320	28	18	46	28
	2655	82	94	15	8.4	26	18
	2741	260	450	71	8.1	148	140
	2763	76	100	32	7.5	28	21
	2780	48	58	20	6.3	97	90
	2878	12	14	18	26	97	71
	2883	200	210	4.6	8.3	72	64
	2904	59	61	5.0	40	79	39
	2919	110	100	-6.0	5.1	53	48
	2928	160	190	16	4.0	11	7.0
	3011	84	110	29	20	188	168
	3025	*1.5*	59	3926	9.1	145	136
	3037	320	330	2.6	78	192	114
	3084	110	190	77	6.0	103	97
	3115	66	89	34	6.9	188	181
	3142	470	590	27	4.8	157	153
	3235	110	130	12	6.5	27	20
	3323	120	140	19	10	26	16

	3331	690	710	2.3	79	130	52
	3372	200	410	110	4.7	51	47
	3429	250	210	-16	30	47	17
	3443	180	280	52	9.9	75	65
	3448	510	660	30	14	172	158
	3472	190	290	54	34	198	164
	3517	210	450	108	5.2	61	56
	3519	480	350	-27	31	51	20
	3579	*4.4*	*4.9*	11	6.1	32	26
	3661	52	63	23	3.6	123	120
	3666	67	80	20	9.6	49	40
	3732	160	190	16	8.3	70	62
	3735	51	13	-75	3.7	66	63
	3742	42	49	17	5.2	52	47
	3791	1000	1600	51	15	137	121
	3796	89	150	64	5.3	128	123
	3836	530	540	2.1	14	145	130
	3840	780	1400	84	4.5	52	47
	3855	450	740	64	292	478	186
	3893	35	71	101	7.4	100	92
	3906	370	420	13	20	114	95
	3918	51	72	41	5.0	158	153
	3927	180	360	97	12	69	57
	3928	33	49	48	14	24	11
	3980	190	250	28	14	38	23
	4008	21	34	65	4.7	31	26
	4010	180	230	29	7.6	36	28
	4041	140	150	6.9	60	77	17
	4068	630	600	-5.2	37	60	23

Table S2: Individual accuracy values for diazepam and nordiazepam mixed effect models; "mathematically calculated concentrations of t1" were calculated based on t2 analytically measured concentration by taking into account Δt; accuracies compare t1-measured concentration with t1-mathematically calculated concentration.

Drug (of abuse)	Case number	Δt (t1-t2) [h]	Measured concentration [ng/mL]		Mathematically calculated concentration [ng/mL]	Accuracy [%]
			t2	t1	t1	
Diazepam	2308	-120	18	30	25	120
	2330	-244	24	29	50	58
	2339	-77	26	41	33	125
	2371	-119	14	4.5	20	22
	2451	-57	96	153	114	134
	2498	-136	239	340	357	95
	2525	-77	111	78	140	56
	2656	-150	5.0	5.7	7.8	73
	2752	-123	142	500	204	246
	2789	-21	53	71	56	127
	2817	-53	147	305	172	178
	2895	-44	82	85	94	91
	2924	-26	10	10	11	93
	2927	-145	131	57	201	28
	2978	-93	24	31	31	98
	2979	-70	51	91	63	143
	3137	-38	132	125	148	84
	3227	-147	67	115	104	111
	3249	-82	58	62	75	83
	3375	-109	24	48	33	144
	3489	-110	54	140	75	187
	3524	-46	80	150	92	163
	3547	-39	64	66	72	92
	3617	-77	23	41	29	141
	3760	-199	215	249	388	64
	3824	-16	39	44	41	107
	3891	-21	22	34	24	144
	4029	-29	265	392	289	136
Nordiazepam	2451	-57	32	50	38	129
	2526	-151	101	71	160	44
	2570	-194	148	302	267	113
	2581	-34	14	15	16	94
	2622	-70	132	78	163	48
	2788	-22	63	62	67	93
	2789	-21	21	31	23	134
	2811	-66	27	43	33	131
	2843	-10	37	29	38	77
	2970	-88	77	99	101	98
	3174	-154	17	39	27	146
	3227	-147	33	56	51	109
	3249	-82	82	111	105	106

	3448	-158	177	485	287	169
	3537	-74	45	84	57	148
	3568	-107	241	253	334	76
	3573	-23	124	119	133	89
	3617	-77	29	52	37	140
	3676	-135	75	84	113	74
	3787	-136	11	15	16	96
	3821	-47	78	74	90	82
	3824	-16	57	66	60	109
	3888	-115	95	167	134	124
	3900	-64	148	172	179	96
	3954	-121	70	117	101	116
	3985	-72	36	49	44	111

Table S3: List of lower limits of quantification (LLOQ) for the discussed drugs (of abuse) including their corresponding calibration ranges (^{12}C and ^{13}C calibration)

Drug (of abuse)	Drug class	LLOQ [ng/mL]	Calibration range [ng/mL]
**Diazepam**	Benzodiazepine	5.3	5.3 – 10’500
**Nordiazepam**	Benzodiazepine	9.4	9.4 – 18’750
**Morphine**	Opioid	2.0	2.0 – 16’000
**Codeine**	Opioid	2.0	2.0 – 16’000
**Citalopram**	Antidepressant	5.0	5.0 – 9’600
**Mirtazapine**	Antidepressant	5.0	5.0 – 4’800
